# Overnight smartphone use: A new public health challenge? A novel study design based on high-resolution smartphone data

**DOI:** 10.1371/journal.pone.0204811

**Published:** 2018-10-16

**Authors:** Naja Hulvej Rod, Agnete Skovlund Dissing, Alice Clark, Thomas Alexander Gerds, Rikke Lund

**Affiliations:** 1 Section of Epidemiology, Department of Public Health, University of Copenhagen, Øster Farigmagsgade 5, DK, Copenhagen K, Denmark; 2 Copenhagen Stress Research Centre, Copenhagen, Denmark; 3 Section of Social Medicine, Department of Public Health, University of Copenhagen, Øster Farigmagsgade 5, DK, Copenhagen K, Denmark; 4 Section of Biostatistics, Department of Public Health, University of Copenhagen, Øster Farigmagsgade 5, DK, Copenhagen K, Denmark; 5 Center for Healthy Aging, Faculty of Health Sciences, University of Copenhagen, Blegdamsvej 3B, DK, Copenhagen N, Denmark; TNO, NETHERLANDS

## Abstract

**Background:**

Round-the-clock use of smartphones holds a potential for awakenings and/or shorter sleep duration, which may have adverse health consequences. We aim to describe overnight smartphone activity among young adults and to characterize those with smartphone interrupted sleep in terms of sleep impairment and mental and physical health indicators.

**Methods:**

We use unique objective high-resolution information on timing of smartphone activity (based on >250,000 phone actions) continuously monitored over a four-week period among 815 young adults combined with indicators of mental and physical health.

**Results:**

We find substantial overnight smartphone activity. More than 12% had smartphone activity in the middle of the night (3 to 5 hours after self-reported bedtime) and 41% had smartphone interrupted sleep on at least one weekday during a 4-week period. Those with frequent smartphone interrupted sleep had on average 48 minutes shorter self-reported sleep duration and higher body mass index, whereas there were no differences in physical or mental health symptoms.

**Conclusions:**

The substantial smartphone activity during bed hours among young adults may pose a public health challenge and especially the relation to overweight warrants close attention.

## Introduction

Bip…zzzz…bip…zzz…bip…zz…bip…z…! Being awakened during sleep is a well-known method used in experimental sleep studies to show adverse health consequences of sleep deprivation and impaired sleep quality, and such experimental studies have rather consistently shown detrimental effects on physiological and mental functioning following sleep interruptions [1,2].

Smartphones became readily available during the noughties and the widespread use of smartphones provides an interesting analogy to experimental sleep studies. Smartphones are easily carried into bed and offer multiple facilities (calling, social networking, texting, gaming, internet etc.), which may disrupt sleep initiation and maintenance. Several studies have previously reported negative effects of technology use on sleep, health and well-being [3–5]. The validity and translation of these findings are, however, limited by the fact that they are based solely on self-reported mobile phone use and do not cover overnight smartphone use. The vast majority of these studies are also conducted among school aged children or adolescents, where parental decisions on technology use play an important role.

Sleep needs vary between individuals, but if sleep is continuously disrupted it is likely to interfere with normal biological restitution during nighttime. Human sleep is composed of rapid eye movement (REM) sleep and gradually deeper stages of non-REM sleep, the deepest of which is called slow wave sleep [1]. This stage is where most of nightly biological restitution takes place. Throughout the night we continuously progress through the different sleep stages in a cyclic manner, with a normal sleep cycle lasting around 90–110 minutes. If sleep is continuously disrupted it may prevent sufficient time in slow wave sleep and biological restitution can be hampered with detrimental effects for future health [6–8].

The massive and increasing 24-hour usage of smartphones, and especially its impact on sleep duration and sleep quality, may raise public health concerns. The underlying assumption of massive smartphone use at night is, however, seldom tested and remains unchallenged. We aim to comprehensively describe overnight smartphone activity in a unique dataset with objective high-resolution information on timing of smartphone activity (including calls, texting, and social networking) in 815 young adults continuously monitored for four weeks. Smartphones are immediately accessible and our hypothesis is that people may react to e.g. incoming text messages even during sleep, which can lead to mental stimulation and interference with normal sleep rhythms. We also aim to characterize people with smartphone interrupted sleep, defined as having less than six consecutive hours without smartphone activity within the self-reported sleep duration, and evaluate how smartphone interrupted sleep relates to sleep duration and quality, body mass index, and physical and mental health symptoms.

## Methods

### Copenhagen social networks study

We used data from the Copenhagen Social Networks Study, which was established to study social activity and behaviors based on continuous monitoring and collection of smartphone data [9]. At enrollment in late August 2013, 3329 undergraduate students at the Technical University of Denmark were invited to participate in the study via their official acceptance letter from the university. 979 (29%) students accepted the invitation, of which 60% were freshmen students. The gender and age distribution of the participants (men: 77%, mean age: 21.6) corresponded well to the distribution among all freshmen enrolled in 2013 at the university (men: 68%, mean age: 21 years). All participants signed an informed consent form. They were given a new smartphone if they inserted their own SIM-card into the phone and responded to a baseline questionnaire containing questions on sleep quality, sleeping hours, and physical and mental health. Responding to the baseline questionnaire was a mandatory part of the enrollment procedure, and the participants either filled in the baseline questionnaire at home before collecting the smartphone, or they were asked to fill in the questionnaire at campus when collecting the smartphone. The smartphone data collection was started as soon as the smartphone was handed out and activated by the participant. The provided smartphone was running customized software continuously recording information on amongst other Facebook activity such as likes and status updates as well as call and text message activity (not content) round-the-clock. A detailed description of the high-resolution smartphone data collection can be seen in Stopczynski et al. 2014 [9]. All data collection was carried out in keeping with regulations from the Danish Data Protection Agency (Approval number: 2012-41-0664). The current study does not require approval by the National Committee on Health Research Ethics by Danish law. As we assumed the sleep patterns over the weekends to be considerably different from weekdays, we only used data from weekdays (Monday through Thursday) from the four-week period starting one week after the participants first activated their smartphone. We excluded the first week of phone use to allow for adjustment to use following receipt of a new phone. We also excluded individuals with no information on self-reported health indicators (N = 59), and with missing phone recordings (N = 105) yielding a total sample of 815 individuals who were included in the analyses.

### Smartphone activity during the sleep period

We recorded the exact timing of smartphone activity from one hour before self-reported bedtime throughout the self-reported sleep period. We were thereby able to determine the proportion who reported to be asleep, but who were in fact active on their smartphones using different features. We recorded each of the following smartphone activities during the self-reported sleep period (as they all require active engagement and thus is expected to interfere with sleep): *Call-activity* was recorded at the time of answered ingoing calls (duration >0 sec) or outgoing calls from the smartphone (irrespective of duration); *SMS-activity* was recorded at the time when an outgoing text message was sent; *Facebook-activity* was recorded at the time of uploaded status-report or ‘liking’ a post on Facebook.

### Smartphone interrupted sleep

Building on the information described above, we were able to determine the longest consecutive passive period (without smartphone-activity) within the self-reported sleep period. We defined smartphone interrupted sleep as having less than six consecutive hours without smartphone activity during self-reported sleep on a weekday, as it is well established in the literature that less than six hours of sleep is related to higher risk of morbidity and mortality [10]. Based on the sixteen weekdays embedded in the four week study period we created an indicator of frequency of smartphone interrupted sleep: Uninterrupted; occasionally interrupted (defined as one to three nights with less than six hours of consecutive inactivity), and frequently interrupted (defined as four or more nights with less than six hours of consecutive inactivity).

### Self-reported sleep measures

*Sleep duration* was calculated based on self-reported information on when students usually go to sleep and wake up in the morning on weekdays (during the past two weeks). *Disturbed sleep* was assessed by the Karolinska Sleep Questionnaire [11], which covers the frequency (from every night = 5 to never = 1) of four symptoms of disturbed sleep: difficulties falling asleep, disturbed/uneasy sleep, repeated awakenings with difficulties falling asleep again and premature awakenings. These four symptoms were combined into a disturbed sleep score reflecting the average frequency of symptoms of disturbed sleep (range 1 to 5).

### Physical and mental health measures

*Self-rated health* was derived from a single SF-36 item on a five-point scale from excellent to poor; *BMI* was determined based on the students’ self-reported height and weight. The students reported whether they during the last two weeks had been bothered (no, a little bothered, very bothered) by the following *physical symptoms*: pain or discomfort in the shoulder/neck, back/loins, legs/knees/hips/joints, or had experienced problems with rapid heartbeat, indigestion, eczema, breathlessness, headache, palpitation or have had a cold. The participants were in the same manner asked about *mental health symptoms*: anxiety, depressive state, sleeping problems, and feeling tired. *Depressive symptoms* were also measured by the Major Depression Inventory [12], which is a self-reported 12-item mood questionnaire. Moderate to severe depressive symptoms were defined as scoring four or five in at least two of the items assessing core symptoms of depression, plus a score of at least three on four of the last seven items assessing accompanying symptoms.

### Analytical methods

Firstly, we report the frequency of smartphone activity during the entire self-reported sleep period on weekdays within a four-week period based on more than 250,000 phone actions (calls, SMS, Facebook). Of these, 19,416 actions occurred during the self-reported sleep period. We categorized self-reported sleep duration into one-hour time-slots, from one hour before self-reported bedtime throughout the self-reported sleep period and counted the number of individuals having defined smartphone activated features at least once during each time-slot on one or more of the 16 weekdays nested within the four-week study period.

Secondly, we calculated the proportions with occasional and frequent smartphone interrupted sleep and characterized these people in terms of age, gender and self-reported sleep measures. Differences in distribution of categorical variables across groups of smartphone interrupted sleep were tested with a chi-squared test, whereas differences in means across groups were tested with an analysis of variance (ANOVA) test. Finally, we assessed the associations between smartphone interrupted sleep and self-rated health, body mass index, physical and mental health symptoms by logistic regression analyses adjusting for gender and age. Tests for trends were based on a Wald test in the logistic regression were each risk factor was included as a linear effect in the model. The measure of smartphone interrupted sleep was defined according to self-reported habitual bed and awakening time. Bedtime may, however, vary from day to day and in a sensitivity analysis, we therefore also used a broader definition where we determined the longest consecutive period without smartphone activity between 9 pm in the evening and 9 am the following morning.

## Results

### Smartphone activity in the sleep period

**Fig 1:**
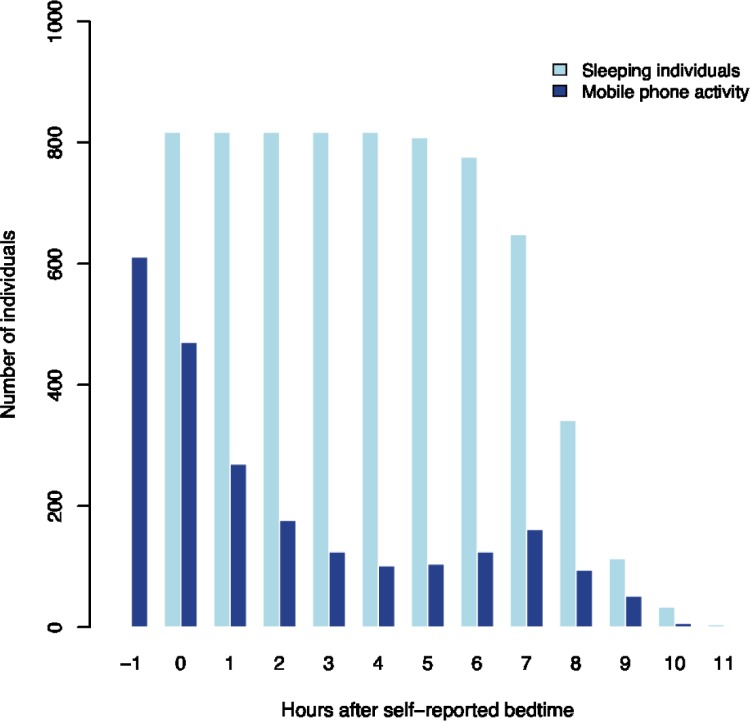
Smartphone activity during self-reported sleeping hours. Number of individuals with at least one mobile phone activity (call, sms, likes, status) in four consecutive weeks (dark blue bars) relative to their self-reported sleeping period (light blue bars). Fridays and weekends are excluded.

**Fig 2:**
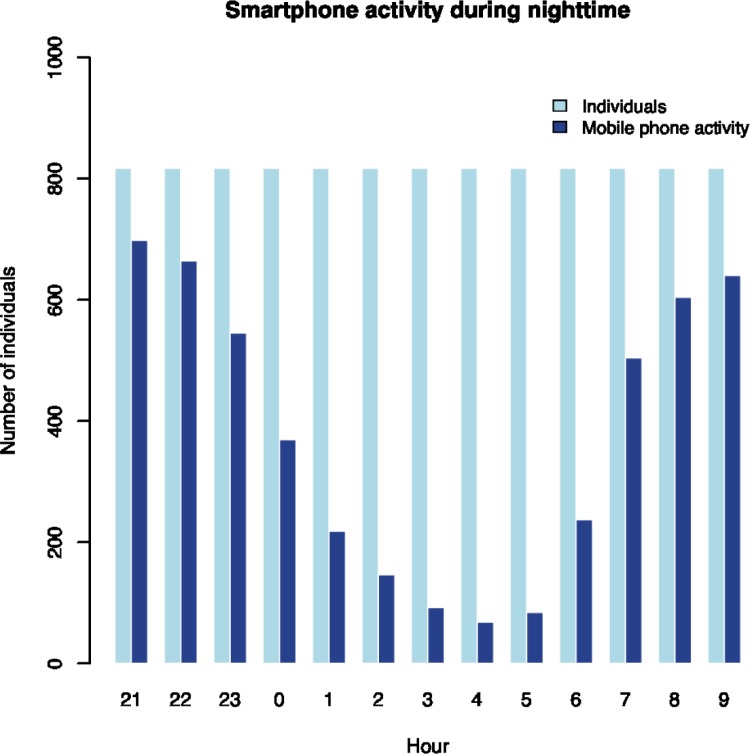
Smartphone activity during nighttime. Number of individuals with at least one mobile phone activity (call, sms, likes, status) in four consecutive weeks (dark blue bars) relative to the hour of the night. The light blue bars show number of individuals. Fridays and weekends are excluded.

The average self-reported sleep duration was 7.6 hours, ranging from 4 hours to 11.5 hours. There was substantial smartphone activity during the self-reported sleep period, as presented in Fig 1. The vast majority (75%) of the participants had smartphone activity in the hour before self-reported bedtime and the proportion with smartphone activity gradually declined during the first 4 to 5 hours of self-reported sleep and then increased again as the waking time approached. One in three had smartphone activity within the first two hours of the self-reported sleep period and the proportion with smartphone activity increased after seven hours of self-reported sleep. While smartphone activity in the hours around bedtime and awakening is expected, it is striking that 12–15 percent also had smartphone activity in the middle of the self-reported sleep period, between 3 and 5 hours after self-reported bedtime. These measures are based on 19,416 smartphone activities during self-reported sleep period among the 815 participants over a four-week period. Interestingly, the majority of these activities were due to outgoing text messages (76.5%), while calls (21.7%) and likes or status updates on Facebook (1.8%) were much less prevalent. In a sensitivity analysis, we assessed the number of people with smartphone activity between 9 pm in the evening and 9 am the following morning, irrespectively of self-reported bedtime (Fig 2). We see an expected pattern with high frequency of smartphone usage in the evening and in the morning, but again it is striking that 8 to 18 percent had smartphone activity in middle of the night (between 2 and 5 am) on weekdays.

### Frequency of smartphone interrupted sleep

**Fig 3:**
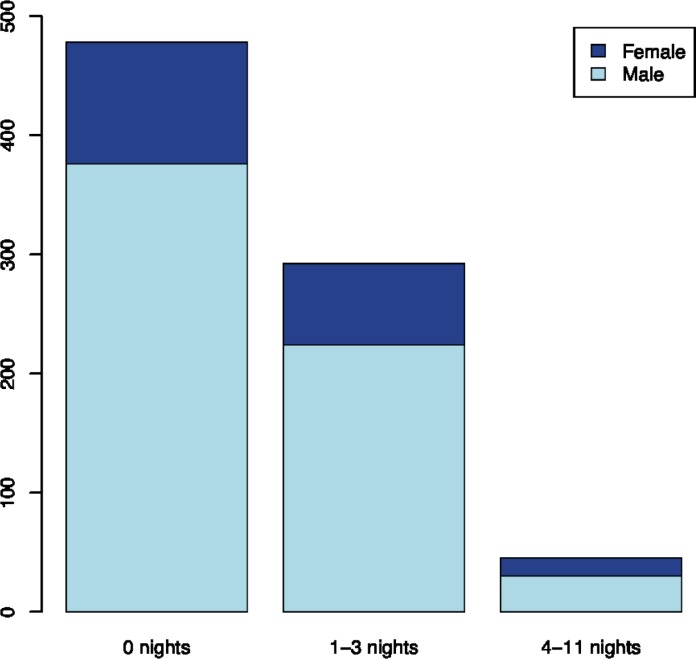
Nights with smartphone interrupted sleep. Distribution of individuals according to gender and number of nights with smartphone interrupted sleep in four consecutive weeks. Fridays and weekends are excluded. Smartphone interrupted sleep was defined as having less than six consecutive hours without smartphone activity during self-reported sleep on a weekday.

More than one third of the study population (41%) experienced one or more days of smartphone interrupted sleep, i.e. less than six hours of smartphone uninterrupted sleep during the self-reported sleep period, on weekdays within a four-week period (16 days). These forty-one percent can be divided into 36% with occasionally interrupted sleep (1 to 3 nights) and 6% with frequently interrupted sleep (4 or more nights) (Fig 3). A slightly higher proportion of women than men had their sleep frequently interrupted by smartphone use. The mean age of the study population is 21.6 years and there were no mean age differences between the groups defined by frequency of smartphone use, as described above. The associations between objective measures of smartphone interrupted sleep and self-reported measures of sleep are presented in Table 1. We found an association between level of interrupted sleep and self-reported sleep length (*P*<0.001). Comparing the average self-reported sleep length across groups with differences in interrupted sleep, we found that those with frequently interrupted sleep on average reported to sleep almost one hour less than those with uninterrupted sleep (7.0 vs. 7.8 hours). Apart from that, we found no clear associations between self-reported sleep and smartphone interrupted sleep. In a sensitivity analysis, we applied a broader definition of smartphone interrupted sleep as less than 6 hours on consecutive smartphone inactivity between 9 pm in the evening and 9 am the following morning. With this broader definition, we found that 15% of the participants have had one or more nights of smartphone interrupted sleep on weekdays during the four-week period.

**Table 1:** Characteristics of the 815 participants in the Copenhagen Social Network Study.

		Smartphone interrupted sleep^a^			
	Total population (n = 815)	Uninterrupted (n = 502)	Occasionally interrupted (n = 273)	Frequently interrupted (n = 40)	P-value
**Socio-demographic measures**					
Women, n (%)	185 (23)	102 (21)	68 (23)	15 (33)	0.17
Age, mean (sd)	21.6 (2.6)	21.6 (2.8)	21.7 (2.5)	21.2 (1.5)	0.52
**Self-reported sleep measures**					
Sleep duration, mean (sd)	7.6 (1.1)	7.8 (1.1)	7.5 (1.0)	7.0 (1.1)	<0.001
Karolinska Sleep Questionnaire items^b^					
Difficulties falling asleep, n (%)	131 (16)	72 (15)	54 (19)	5 (11)	0.20
Disturbed/uneasy sleep, n (%)	86 (11)	53 (11)	29 (10)	4 (9)	0.86
Repeated awakenings, n (%)	32 (4)	17 (4)	14 (5)	1 (2)	0.44
Premature awakenings, n (%)	87 (11)	54 (11)	28 (10)	5 (11)	0.63
Disturbed sleep score^c^, mean (sd)	2.1 (0.7)	2.1 (0.7)	2.1 (0.7)	1.8 (0.7)	0.12

^a^ Smartphone interrupted sleep is defined as less than 6 hours on consecutive smartphone inactivity during self-reported sleep on weekday (Fridays and weekends excluded) over a four week period. Uninterrupted (no smartphone interrupted sleep); occasionally interrupted (defined as one to three nights with smartphone interrupted sleep), and frequently interrupted (defined as four or more nights with smartphone interrupted sleep).

^b^ Reporting ‘almost every night’ or ‘several times a week’ for each item

^c^ The four items of the Karolinska Sleep Questionnair were combined into an index reflecting the average frequency of symptoms (range 1 to 5)

### Smartphone interrupted sleep and health measures

The associations between smartphone interrupted sleep and health measures are presented in Table 2. Frequent smartphone interrupted sleep seemed to be associated with more sub-optimal self-rated health (OR = 1.79; 95% CI: 0.65–4.90), but the estimate was only based on 45 individuals and was not statistically significant. Frequency of smartphone interrupted sleep was associated with being overweight (P = 0.02). Occasionally interrupted sleep was associated with a 1.86 (95% CI: 1.20–2.87) higher odds of overweight compared to those with uninterrupted sleep. A similar association was found for those frequently exposed to smartphone interrupted sleep, although not statistically significant. Apart from these findings, there were no clear differences in physical or mental health symptoms among those with and without smartphone interrupted sleep.

**Table 2:** Associations between smartphone interrupted sleep and health measures among 815 participants in the Copenhagen Social Network Study, adjusted for age and sex.

	Smartphone interrupted sleep OR (95% CI)			P-value for trend
	Uninterrupted	Occasionally	Frequently	
**Sub-optimal self-rated health**	1 (ref)	0.91 (0.49; 1.69)	1.79 (0.65; 4.90)	0.58
**Overweight**	1 (ref)	1.86 (1.20; 2.87)	1.52 (0.61; 3.80)	0.02
Physical symptoms*				
Shoulder/neck	1 (ref)	0.97 (0.71; 1.31)	0.82 (0.43; 1.57)	0.61
Back/loins	1 (ref)	0.85 (0.63; 1.16)	1.12 (0.59; 2.11)	0.65
Lower extremities	1 (ref)	1.08 (0.78; 1.50)	1.12 (0.57; 2.21)	0.61
Indigestion	1 (ref)	0.74 (0.50; 1.09)	0.50 (0.19; 1.30)	0.05
Eczema	1 (ref)	1.14 (0.74; 1.77)	0.72 (0.25; 2.08)	0.97
Cold	1 (ref)	1.19 (0.87; 1.62)	0.74 (0.37; 1.48)	0.84
Stomach ache	1 (ref)	1.28 (0.86; 1.92)	1.54 (0.71; 3.32)	0.14
Headache	1 (ref)	1.08 (0.88; 1.46)	0.99 (0.53; 1.87)	0.76
Breathlessness	1 (ref)	1.54 (0.97; 2.46)	1.48 (0.59; 3.73)	0.09
Palpitation	1 (ref)	1.58 (0.98; 2.54)	1.07 (0.36; 3.16)	0.18
**3 or more physical symptoms**	1 (ref)	1.32 (0.94; 1.86)	0.78 (0.36; 1.70)	0.48
Mental health symptoms*				
Anxiety	1 (ref)	0.88 (0.59; 1.32)	0.90 (0.40; 2.05)	0.57
Depressed	1 (ref)	1.24 (0.87; 1.78)	0.66 (0.27; 1.61)	0.82
Sleeping problems	1 (ref)	1.28 (0.94; 1.74)	1.10 (0.57; 2.10)	0.21
Tiredness	1 (ref)	1.25 (0.91; 1.73)	0.90 (0.47; 1.73)	0.46
**2 or more mental health symptoms**	1 (ref)	1.22 (0.82; 1.83)	0.90 (0.36; 2.22)	0.61
**Moderate to severe depressive symptoms**	1 (ref)	1.22 (0.90; 1.65)	0.95 (0.51; 1.78)	0.43

* Reporting to be some or very bothered by the symptom within the last two weeks.

## Discussion

We find substantial overnight smartphone activity in a unique dataset with objective information on duration and timing of smartphone activity in 815 young adults continuously monitored for four weeks. While smartphone activity around bedtime and awakening is expected, it is striking that more than 12 percent have smartphone activity in the middle of the sleep period and that 41% have smartphone interrupted sleep on at least one weekday during a four-week period. Those with frequently interrupted sleep had shorter self-reported sleep duration and higher body mass index. Apart from this there were no major differences in physical or mental symptoms according to the level of smartphone interrupted sleep.

Technology use during daytime is almost inevitable for societal involvement as for work and social lives. A majority of young adults in a study from Sweden felt that they were expected to be continuously accessible via mobile phone, but that this accessibility was not necessarily perceived as stressful to them.^5^ An auxiliary qualitative study found that this expected accessibility was partly self-embedded as a ‘fear of missing out’[13]. Mobile phone use around bedtime and during sleep hours may, however, affect sleep by various psychological and physiological mechanisms. We found shorter self-reported sleep duration among those with smartphone interrupted sleep, which may be a result of sleep displacement, i.e. sleep is substituted by smartphone use at bedtime or during the sleep period.

There is an extensive literature on bedtime use of electronic devices and how it affects sleep. In a cross-sectional study based on 9,846 adolescents from Norway, 90% of the adolescents reported to use their mobile phone in the hour before going to bed and this was related to self-reported sleep onset latency and sleep deficit [14]. The vast majority of previous studies on mobile phone use and sleep have been conducted in children and adolescents and they have quite consistently shown adverse effects of bedtime technology use on sleep length and sleep quality [4,14–17]. Media use in childhood and adolescence is highly affected by parental control and sleep patterns are vulnerable to developmental changes, e.g. puberty, and results from these studies are therefore not directly transferable to an adult population. One of the few studies in adults include a recent cross-sectional study among 844 Flemish adults, in which self-reported bedtime mobile phone use was associated with poorer sleep quality, more fatigue and insomnia symptoms [18]. We found that overnight smartphone use was related to shorter sleep duration, but not indicators of sleep quality.

The artificial bright light exposure from smartphones can delay release of the sleep hormone melatonin [19], which may explain problems with sleep initiation associated with bedtime use of electronic devises observed in several studies [4]. Mobile phones transmit and receive signal by electromagnetic fields and exposure to electromagnetic radiation immediately prior to sleep initiation have been shown to decrease rapid eye movement (REM) sleep latency [20]. Although we found no clear associations between smartphone disrupted sleep and problems falling asleep, the vast majority of the study participants in the present study used their smartphone around bedtime and almost one in six persons had frequent problems falling asleep.

While smartphone use is becoming a bedtime ritual for many people, disruption of sleep during bed hours by smartphones or other electronic devices may need to be treated as a separate problem. Earlier findings from a Swedish study on young adults published in 2011 showed that 13% of men and 17% of women reported to be awakened at least a few times per month by their mobile phone [5]. In the current study, we show that overnight smartphone activity is quite common in young adults, with more than one third of the young adults in our study having experienced smartphone interrupted sleep on weekdays within a four-week period. While we cannot know whether the user was awakened by the smartphone, or whether (s)he was awake for another reason and therefore chose to use the phone, this still deserves public health attention.

We found no clear relation between objectively measured smartphone interrupted sleep and self-reported mental health symptoms or depressive symptoms. These findings are in line with those of a previous study among young adults in Sweden, where there was no clear relation between reporting to be awakened by mobile phone during nighttime and mental health symptoms one year after baseline [5]. This lack of association is surprising given the close interrelation between impaired sleep and depression observed in other studies [21]. A hypothetical explanation may be that the opportunities for social interaction and support provided by the mobile phone may counteract some of the negative influences of frequent use during nighttime.

Short sleep duration is a well-known risk factor for overweight and obesity [22]. Sleep loss can lead to metabolic dysfunctioning through hyperactivation of the HPA axis and changes in the neuroendocrine response [23]. In an experimental study, sleep restriction was found to be associated with elevated levels of the appetite-regulating hormone ghrelin and increased calorie intake from snacks and sweets [24]. In accordance to the proposed mechanisms, we found smartphone interrupted sleep to be associated with shorter sleep duration and higher body mass index. This is an interesting finding in light of the parallel increase in smartphone use and obesity in adult populations across the western world. Previous studies on mobile phone use and obesity also provide some support for a relationship, although they have been limited to children/adolescents and have solely been based on self-reported measures of mobile phone use [3‚ 25].

### Weaknesses and strengths

The present study is cross-sectional in nature making it difficult to separate cause from effect. Although we assume smartphone use to be a source of sleep interruption, the smartphone may merely be used as a sleeping aid or to counteract boredom among those with sleep problems. To counteract the problem of reversed causation one might want to limit the smartphone use to external sources of disruption such as incoming calls and text messages. This distinction is, however, partly meaningless with the current pattern of smartphone use, as incoming messages and calls can be ‘muted’ and not received until the morning. It would potentially also lead to a severe underestimation of the level of nightly interruption.

The sleep period was defined based on self-reported information and we did not have information on actual sleeping hours during the four-week period of interest. This could potentially lead to an overestimation of smartphone interrupted sleep if some of the participants shifted the time when they went to bed and woke up in the morning. In a sensitivity analysis, we applied a broader definition of smartphone interrupted sleep as less than 6 hours of consecutive smartphone inactivity between 9 pm in the evening and 9 am the following morning, to address this concern. With this broader definition we still found that 15% of the participants have had one or more nights of smartphone interrupted sleep on weekdays during the four-week period.

We only had information on smartphone use (including some but not all activities), which may have resulted in a general underestimation of the scope of the problem, as sleep may also be interrupted by other devices including TV viewing, computer or tablet use, and gaming. In our data, 41% of the young adults experienced one or more days of smartphone interrupted sleep, i.e., less than six hours of smartphone uninterrupted sleep on weekdays during a four-week period. This number is only a lower bound and would likely be higher if we had been able to monitor the use of other technological devices or additional smartphone activities, e.g., gaming, streaming of film and television during bed hours. The study was conducted among young adults attending a technical university, and the results may not be directly transferable to older age groups where smartphone use is less embedded in social life.

The participation rate was quite low and one may be concerned about the impact of the selection mechanisms into the study. We unfortunately do not have detailed information on the characteristics of those who did not participate in the study, but the age and gender distribution corresponded well to all freshmen students at the university. One might assume that those who participated in the study were more interested in technology and thus more likely to have a high frequency smartphone use, also at night time, which may have led to an overestimation of the prevalence of smartphone interrupted sleep in the study participants. On the other hand, one may also conjecture that those with sleep problems, mental or physical symptoms would probably be less likely to participate in the study, which is likely to cause the associations between smartphone interrupted sleep and health problems to be underestimated.

In contrast to previous studies, the main advantage of the objective high-resolution data from the Copenhagen Social Network Study is that it provides a comprehensive and objective measure of the study participants’ smartphone use during the entire sleep period. Previous studies have been severely limited by common method bias, i.e. mobile phone use, sleep and health measures have all been measured by self-report, which will often lead to an overestimation of the effect. We propose that future studies on health consequences of smartphone interrupted sleep include objective, high-resolution smartphone data together with other methods (e.g. self-reports on being awakened by their phone) that combined will provide us with accurate measures of smartphone interrupted sleep.

## Conclusions

We document substantial overnight smartphone activity in young adults, but it is unclear whether this smartphone activity is causing sleep interruption or if it is used as an entertainment device among those with sleep impairment due to other causes. It is, however, striking that more than one third of the young adults in the study had smartphone interrupted sleep during at least one weekday within a four-week period. Those with frequently interrupted sleep had shorter self-reported sleep duration and higher body mass index. There were no clear relations to other physical or mental health symptoms. Improving sleep hygiene through better management of technology, including advice to set limits for accessibility during the sleep period, may improve health and well-being in the general population.

## References

[1] Lockley SW. Principles of sleep-wake regulation In: Cappuccio FP, Miller MA, Lockley SW, editors. Sleep, health and society. From aetiology to public health. New York: Oxford University Press; 2010 9–34.

[2] Marshall NS, Stranges S. Sleep duration:risk factor or risk marker for ill health? In: Cappuccio FP, Miller MA, Lockley SW, editors. Sleep, health and society. From etiology to publiuc health. New York: Oxford University Press; 2010 35–49.

[3] Arora T, Hussain S, Hubert Lam KB, Lily YG, Neil TG, Taheri S. Exploring the complex pathways among specific types of technology, self-reported sleep duration and body mass index in UK adolescents. *Int J Obes (Lond)* 2013; 37(9):1254–1260. 23295500 10.1038/ijo.2012.209

[4] Cain N, Gradisar M. Electronic media use and sleep in school-aged children and adolescents: A review. *Sleep Med* 2010; 11(8):735–742. 10.1016/j.sleep.2010.02.006 20673649

[5] Thomee S, Harenstam A, Hagberg M. Mobile phone use and stress, sleep disturbances, and symptoms of depression among young adults—a prospective cohort study. *BMC Public Health* 2011; 11:66 10.1186/1471-2458-11-66 21281471 PMC3042390

[6] Rod NH, Vahtera J, Westerlund H, Kivimaki M, Zins M, Goldberg M et al Sleep disturbances and cause-specific mortality: Results from the GAZEL cohort study. *Am J Epidemiol* 2011; 173(3):300–309. 10.1093/aje/kwq371 21193534 PMC3105272

[7] Tasali E, Leproult R, Spiegel K. Reduced sleep duration or quality: relationships with insulin resistance and type 2 diabetes. *Prog Cardiovasc Dis* 2009; 51(5):381–391. 10.1016/j.pcad.2008.10.002 19249444

[8] Cappuccio FP, D'Elia L, Strazzullo P, Miller MA. Sleep duration and all-cause mortality: a systematic review and meta-analysis of prospective studies. *Sleep* 2010; 33(5):585–592. 20469800 10.1093/sleep/33.5.585 PMC2864873

[9] Stopczynski A, Sekara V, Sapiezynski P, Cuttone A, Madsen MM, Larsen JE et al Measuring large-scale social networks with high resolution. *PLoS One* 2014; 9(4):e95978 10.1371/journal.pone.0095978 24770359 PMC4000208

[10] Watson NF, Badr MS, Belenky G, Bliwise DL, Buxton OM, Buysse D et al Joint Consensus Statement of the American Academy of Sleep Medicine and Sleep Research Society on the Recommended Amount of Sleep for a Healthy Adult: Methodology and Discussion. *Sleep* 2015; 38(8):1161–1183. 10.5665/sleep.4886 26194576 PMC4507722

[11] Akerstedt T, Knutsson A, Westerholm P, Theorell T, Alfredsson L, Kecklund G. Sleep disturbances, work stress and work hours: a cross-sectional study. *J Psychosom Res* 2002; 53(3):741–748. 12217447 10.1016/s0022-3999(02)00333-1

[12] Bech P, Rasmussen NA, Olsen LR, Noerholm V, Abildgaard W. The sensitivity and specificity of the Major Depression Inventory, using the Present State Examination as the index of diagnostic validity. *J Affect Disord* 2001; 66(2–3):159–164. 11578668 10.1016/s0165-0327(00)00309-8

[13] Thomee S, Dellve L, Harenstam A, Hagberg M. Perceived connections between information and communication technology use and mental symptoms among young adults—a qualitative study. *BMC Public Health* 2010; 10:66 10.1186/1471-2458-10-66 20152023 PMC2836296

[14] Hysing M, Pallesen S, Stormark KM, Jakobsen R, Lundervold AJ, Sivertsen B. Sleep and use of electronic devices in adolescence: results from a large population-based study. *BMJ Open* 2015; 5(1):e006748 10.1136/bmjopen-2014-006748 25643702 PMC4316480

[15] Arora T, Broglia E, Thomas GN, Taheri S. Associations between specific technologies and adolescent sleep quantity, sleep quality, and parasomnias. *Sleep Med* 2014; 15(2):240–247. 10.1016/j.sleep.2013.08.799 24394730

[16] Munezawa T, Kaneita Y, Osaki Y, Kanda H, Minowa M, Suzuki K et al The association between use of mobile phones after lights out and sleep disturbances among Japanese adolescents: a nationwide cross-sectional survey. *Sleep* 2011; 34(8):1013–1020. 10.5665/SLEEP.1152 21804663 PMC3138156

[17] Van den Bulck J. Adolescent use of mobile phones for calling and for sending text messages after lights out: results from a prospective cohort study with a one-year follow-up. *Sleep* 2007; 30(9):1220–1223. 17910394 10.1093/sleep/30.9.1220 PMC1978406

[18] Exelmans L, Van den Bulck J. Bedtime mobile phone use and sleep in adults. *Soc Sci Med* 2016; 148:93–101. 10.1016/j.socscimed.2015.11.037 26688552

[19] Wood AW, Loughran SP, Stough C. Does evening exposure to mobile phone radiation affect subsequent melatonin production? *Int J Radiat Biol* 2006; 82(2):69–76. 10.1080/09553000600599775 16546905

[20] Loughran SP, Wood AW, Barton JM, Croft RJ, Thompson B, Stough C. The effect of electromagnetic fields emitted by mobile phones on human sleep. *Neuroreport* 2005; 16(17):1973–1976. 16272890 10.1097/01.wnr.0000186593.79705.3c

[21] Weich S. The epidemiology of sleep and depression Sleep, health and society. From aetiology to public health. New York: Oxford University Press; 2010 178–190.

[22] Cappuccio FP, Taggart FM, Kandala NB, Currie A, Peile E, Stranges S et al Meta-analysis of short sleep duration and obesity in children and adults. *Sleep* 2008; 31(5):619–626. 18517032 10.1093/sleep/31.5.619 PMC2398753

[23] Hirotsu C, Tufik S, Andersen ML. Interactions between sleep, stress, and metabolism: From physiological to pathological conditions. *Sleep Sci* 2015; 8(3):143–152. 10.1016/j.slsci.2015.09.002 26779321 PMC4688585

[24] Broussard JL, Kilkus JM, Delebecque F, Abraham V, Day A, Whitmore HR et al Elevated ghrelin predicts food intake during experimental sleep restriction. *Obesity (Silver Spring)* 2016; 24(1):132–138. 26467988 10.1002/oby.21321 PMC4688118

[25] Chahal H, Fung C, Kuhle S, Veugelers PJ. Availability and night-time use of electronic entertainment and communication devices are associated with short sleep duration and obesity among Canadian children. *Pediatr Obes* 2013; 8(1):42–51. 10.1111/j.2047-6310.2012.00085.x 22962067

